# Application of artificial intelligence for the prediction of amyloidoses

**DOI:** 10.4103/NRR.NRR-D-25-00482

**Published:** 2025-09-03

**Authors:** Valentin Gonay, Michael P. Dunne, Andrey V. Kajava

**Affiliations:** CRBM UMR 5237 CNRS, Université Montpellier, Montpellier, France; PROTERA SAS, Paris, France

Amyloidosis is a group of diseases caused by the abnormal accumulation of amyloid fibrils (misfolded protein aggregates) in various human tissues. These amyloid deposits can interfere with normal organ function and lead to severe health issues. Amyloid plaque formation in the brain is linked to neurodegenerative diseases, including Tau and Aβ plaques in Alzheimer’s disease, α-synuclein plaques in Parkinson’s disease, and huntingtin plaques in Huntington’s disease. Although this condition is typically associated with aging, certain mutations in amyloid-forming proteins can trigger early-onset amyloidosis (Hatami et al., 2017). Given its progressive and life-threatening nature, early detection and treatment are crucial.

**Figure 1:**
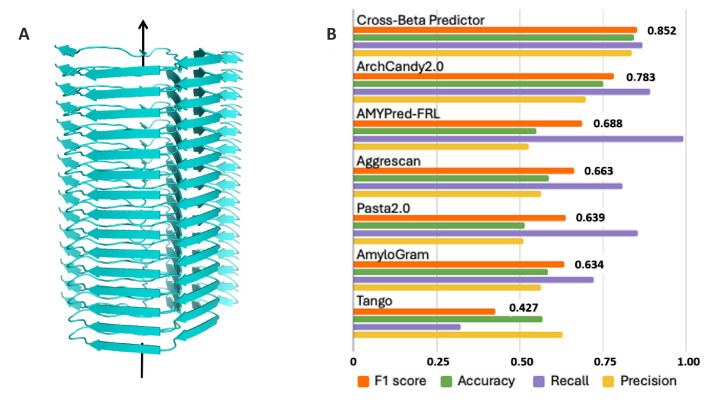
Structural representation of a typical amyloid fibril and benchmarking of amyloidogenicity prediction tools. (A) Lateral view of a typical cross-β structure of an amyloid fibril with parallel and in-register arrangement of the chains formed by the α-synuclein [*Homo sapiens*] (generated from PDB entry 6CU7). The arrow shows the fibril’s axis. Known cross-β amyloids were compiled into the new Cross-Beta DB database, accessible via the website (https://bioinfo.crbm.cnrs.fr/index.php?route=databases&tool=34). Created with PyMOL (Schrödinger, LLC). (B) Performance of various tested predictors of protein amyloidogenicity. The ML-based Cross-Beta predictor outperforms existing computational tools.

Proteins in the amyloid fibrils consistently adopt a characteristic “cross-β” structure, in which polypeptide chains in β-conformation are aligned perpendicularly to the fibril axis (Steven et al., 2016; **Figure 1A**). Numerous studies suggest that a protein’s amino acid sequence determines its amyloid-forming potential. Given this, along with advancements in genetic sequencing, there is a strong foundation for applying computational tools for the early diagnosis of amyloidosis. As a result, since the early 2000s, several bioinformatics teams have developed such computational tools with varying degrees of success (e.g., Fernandez- Escamilla et al., 2004; Ahmed et al., 2015; Burdukiewicz et al., 2017; Charoenkwan et al., 2022).

Recently, inspired by the growing power of artificial intelligence (AI), scientists have begun exploring effective ways to apply machine learning (ML) to predict protein amyloidogenicity (Burdukiewicz et al., 2017; Charoenkwan et al., 2022). However, there has also been widespread skepticism about the suitability of AI methods for this task, largely due to the limited availability of experimentally confirmed data on amyloid-forming proteins. The common perception that ML requires massive datasets stems from the current emphasis on large-scale, unsupervised models and deep neural networks. This trend may overshadow the potential of supervised models, which can perform effectively even with smaller, well-curated datasets. Therefore, we focused our efforts on identifying suitable ML approaches for predicting amyloidogenicity by using supervised learning models, such as decision trees and random forests, which are better suited to problems involving limited data. To address this challenge, we also placed strong emphasis on refining and curating the dataset. As a result, we developed an ML-based predictor of amyloidogenicity that demonstrated superior performance compared to existing methods (Gonay et al., 2025; **Figure 1B**).

However, the path to this result was not straightforward. Our initial attempts to predict naturally occurring amyloids using existing datasets of amyloid-forming proteins and peptides yielded only moderate results with supervised learning models. Comparable outcomes were observed when applying previously developed ML models (Burdukiewicz et al., 2017; Charoenkwan et al., 2022). This prompted us to assess the quality of the datasets of amyloid-forming proteins and peptides used to train ML models. Our analysis of existing databases revealed a mix of protein aggregates, including amorphous, globular, and α-helical structures, not associated with typical cross-β amyloids (Rawat et al., 2020). These databases grouped *in vitro* amyloids, formed under extreme, non-physiological conditions, with *in vivo* fibrils, without distinguishing between the two. Since computational predictors primarily aim to detect amyloids *in vivo*, it is essential to have a database focused solely on the naturally occurring cross-β amyloid fibrils.

Recognizing the crucial importance of not only the volume but also the quality of data used for training ML models, we made significant efforts to refine the dataset, focusing exclusively on cross-β amyloids formed *in vivo*. This led to the construction of a new Cross-Beta DB database (Gonay et al., 2025), featuring “denoised” data. To select entries, we used the following criteria: (i) the amyloid is naturally-occurring (functional or disease-related), (ii) localization of amyloidogenic region (AR) within the protein is known and (iii) the amyloid structure has the cross-β arrangement. The key element of entries in Cross-beta DB was the AR, rather than the complete protein sequence. It turns out that in most naturally occurring cases, including both disease-associated and functional amyloids, the amyloid-forming regions contain structural elements known as β-arches (Kajava et al., 2010) and span more than 15 residues. All short peptides, approximately six residues long, that form amyloid fibrils *in vitro* but not *in vivo* were excluded from the dataset. The current Cross-Beta DB contains 115 unique entries of ARs (https://bioinfo.crbm.cnrs.fr/tools/Cross-Beta-DB/index_DB.php). As the number of proteins known to form amyloids *in vivo* continues to grow, we plan to update the database regularly. To harness community contributions, we have also created a web-based platform for users to add data to the database.

To train our ML models, we created a non-redundant positive dataset of 61 representative amyloid-forming sequences by clustering all Cross-Beta DB entries. A non-redundant negative dataset was constructed from the DisProt database using the same procedure, containing 268 representative sequences known to be soluble (Aspromonte, 2024). We used these datasets to train and benchmark our Cross-Beta predictor. We also made the data sets publicly available as ready-to-use resources for training and benchmarking new AI-based methods for amyloidogenicity prediction. Our Cross-Beta predictor demonstrated superior performance compared to existing methods (**Figure 1B**).

Thus, we draw the following conclusion from this work: ML methods can be successful even when the positive and negative datasets contain fewer than a hundred samples each. For such tasks, supervised learning models like decision trees or random forests are well suited, provided the datasets contain high-quality, “denoised” data. This provides a basis for applying ML approaches to address additional challenges associated with protein aggregation. One key problem is co-aggregation (or cross-seeding) of amyloid-forming proteins (Burdukiewicz et al. 2023). The number of known cases is increasing, highlighting its potential role in the etiology of certain human amyloidoses. For instance, microbial proteins can trigger the formation of amyloids in human proteins, potentially leading to amyloidosis (Semerdzhiev et al., 2021). Currently, data on protein co-aggregation are limited. However, by applying a similar approach to the one used in developing the Cross-Beta predictor, ML-based methods could help address this problem.

Another challenge is predicting proteins involved in liquid–liquid phase separation (LLPS). Similar to existing amyloid databases, LLPS protein databases require careful curation, as these proteins play distinct roles in the process. Scaffold proteins drive LLPS and are essential for the formation and maintenance of phase-separated condensates. In contrast, modulator proteins regulate LLPS without actively driving it, either promoting or inhibiting phase separation. Client molecules, in contrast, are merely recruited into pre-formed condensates. Our preliminary analysis suggests that it is possible to obtain a denoised dataset of condensate-forming proteins with a sufficient number of samples. This will enable us to develop an effective LLPS predictor by applying the approach described in Gonay et al. (2025).

**Figure 2:**
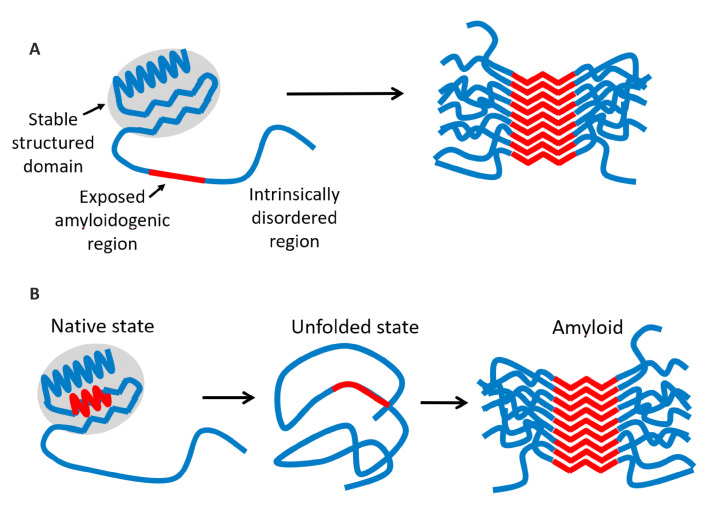
A schematic representation of two amyloidogenesis pathways. (A) The protein’s native conformation contains an amyloidogenic region (in red) within an intrinsically disordered region, making it readily accessible for amyloid formation. (B) The protein’s native conformation harbors an amyloidogenic region (in red) within a stable, structured domain, where it remains buried. To enable amyloid formation, the protein must first undergo unfolding.

The Cross-Beta predictor identifies ARs in proteins, which is essential but not always sufficient for predicting amyloidosis. If a protein’s AR lies within a stable 3D structure, it may be buried and inaccessible for amyloid formation unless the protein undergoes unfolding (**Figure 2**). Therefore, when ARs are located within structured domains rather than intrinsically disordered regions, evaluating native structure stability becomes critical. This is particularly important in hereditary amyloidoses, where missense mutations may destabilize the native fold. While many amyloidosis-associated proteins such as Aβ in Alzheimer’s disease, α-synuclein in Parkinson’s disease, and huntingtin AR in Huntington’s disease are intrinsically disordered (**Figure 2A**), others, such as those in hereditary lysozyme, apolipoprotein A-I, fibrinogen Aα-chain amyloidosis, and ATTR cardiomyopathy, are natively folded (**Figure 2B**). Thus, predicting amyloidosis in these proteins requires the use of both amyloidogenicity predictors and tools that assess the stability of the native protein structure.

Recently, we tackled this issue by analyzing mutations in transthyretin (TTR) associated with hereditary cardiomyopathy and proposed a ML-based approach to distinguish between mutations that lead to amyloidosis and those that do not (Pyankov et al., 2025). Under native conditions, TTR spontaneously folds into a stable globular structure, which then assembles into a homo-tetramer with a well-characterized 3D structure. By analyzing datasets of mutations associated and not associated with hereditary ATTR cardiomyopathy, we examined transthyretin mutants using amyloidogenicity predictors. Additionally, to assess the impact of these mutations on tetramer stability, we employed structural stability predictors. Both in silico results and experimental studies on ATTR-related mutations suggest that structural destabilization increases the likelihood of mutated TTR forming amyloids. However, our analysis showed that calculated ΔΔG values of the destabilization alone are insufficient for accurately predicting ATTR-causing mutations. To improve prediction accuracy, we integrated ΔΔG calculations with additional features into ML algorithms. Since the number of known mutations associated and not associated with hereditary ATTR cardiomyopathy is limited, similar to the case of naturally occurring amyloids and LLPS proteins, we chose to apply supervised ML-models previously used in the development of the Cross-Beta predictor. As a result, we developed an ML model, SDAM-TTR, which demonstrates considerable predictive power in identifying mutations linked to ATTR cardiomyopathy. This strengthens the prospects of improved predictions of unknown mutations associated with other hereditary amyloidoses, such as hereditary lysozyme amyloidosis, apolipoprotein A-I amyloidosis, and fibrinogen Aα-chain amyloidosis, where the amyloid-forming protein initially adopts a stable 3D structure and must unfold to form amyloid fibrils (Pyankov et al., 2025).

In conclusion, through several examples, we demonstrated that despite the limited data on amyloid-forming proteins, AI-based methods can be successfully applied. This is especially true when preceded by comprehensive curation of the positive and negative datasets used for training and benchmarking. The curation must be enhanced by considering conditions such as pH, temperature, concentration, and post-translational modifications under which proteins form or fail to form amyloid fibrils and other types of aggregates. It is important to note that developing a high-performing ML model requires carefully designing features that align with the input data and the specific problem at hand. This process is neither straightforward nor fully automated; in many cases, it relies on human expertise. From a long-term perspective, the application of ML in protein aggregation research looks promising, as the growing number of documented protein-aggregating sequences, linked to both amyloids and amorphous condensates, will drive continuous improvements in the accuracy and predictive power of ML models.

*This work was supported in part by a CIFRE PhD fellowship through the “Association Nationale de la Recherche et de la Technologie” (ANRT) program, in collaboration between PROTERA and “Center National de la Recherche Scientifique” (CNRS) to VG, by EU COST Action ML4NGP CA21160 to VG and AVK, by the Fondation pour la Recherche Médicale (grant MND202310017898) to AVK and by the AFM-Téléthon (grant n° 28988) to AVK.*
